# A match made in heaven - “Indian matchmaking” in contemporary times

**DOI:** 10.3389/fsoc.2022.684994

**Published:** 2022-09-02

**Authors:** Debjyoti Ghosh

**Affiliations:** Postdoctoral Researcher, Department of Sociology, University of Pretoria, Pretoria, South Africa

**Keywords:** postfeminism, feminism, subcontinent, diaspora, femininity, media, women, matchmaking

## Abstract

The Indian subcontinent is ubiquitous with some social factors such as caste, gender (discrimination), poverty. One particular factor that has taken up the imaginations of the Netflix-watching audience of late is the practice of arranged marriages. A series called *Indian Matchmaking* catapulted the notion of arranged marriages into the drawing rooms of both people who are highly aware of the notion (probably having been through it themselves), as well as people who have a very vague idea about it. Nevertheless, it has become a highly talked about television show across the Anglophone world. A little before its release, another English-language reality show, *What the Love! with Karan Johar* was released by Netflix. This explores the world of romantic connections with a few chosen people from India. While placing itself on the opposite side of the spectrum when compared to *Indian Matchmaking*, in many ways, it lends itself to similar tropes, albeit under a progressive garb. This paper delves into the portrayal of people from India or of Indian origin in the reality shows *Indian Matchmaking* and *What the Love! with Karan Johar*. I examine the two shows through the lens of postfeminism and how, while raising several social issues that plague Indian society, both citizens and the diaspora, they inadvertently propagate a certain self-policing and conservatism that people, particularly women, are expected to adhere to.

## Introduction—Ever thine, ever mine, ever each other's^1^ - Beethoven.

It was 2001. *Monsoon Wedding* came out in the cinemas in India, a primarily English-language movie by Nair (2001). It pushed multiple boundaries on issues that were seldom clubbed together in the same script—arranged marriages, broken hearts, child sexual abuse, incest, women utilizing their agency within the constructs of an Indian family. Primarily, though, it is a movie about an upper-middle class family based in New Delhi, planning an Indian wedding—from taking loans to make ends meet, making economies, balancing relatives, emotions, loyalties and betrayal, and ambitions of moving away from India.

However, it wasn't a typical Bollywood song and dance movie. What made the movie truly stand out was the way it portrayed arranged marriages in India and how it challenged western prejudices against the practice. Perhaps, this was one of the first forays of bringing arranged marriages into the twenty-first century for the anglophone audience across the world. Still, when Nair made the film, she probably never thought that Indian weddings, particularly arranging love and marriage, would take over the small screen in the way it has today—that too as reality TV shows.

Online viewing platforms like Netflix have brought soaps, reality television, cinema from across the world onto one global platform. While many of these shows and cinemas are often in their vernacular languages, there is a plethora of English-language shows. While Indian matrimonial columns were brought into the digital age as early as 1997, for Indian matchmaking to become a part of international reality television, we had to wait for two more decades. *Indian Matchmaking* (Mundhra et al., 2020) and *What the Love! with Karan Johar* (Vagal, 2020)^2^ are two reality shows (among others) that showcase Indian or Indian-origin people, and their tribulations in trying to get into relationships or dealing with their existing relationships. Neither of these shows is new in their genres, but as shows that are both in the English language and entirely portraying an Indian or Indian-origin cast, they are unique.

This paper aims at examining the presence of postfeminism in India that is showcased commercially, and how the shows spoken of above have contributed toward an image of middle-class cis-bodied Indian/Indian origin people without going into the intricacies of the complexities of Indian society, and sidelining how the population constantly live at the intersections of different social constructions.

I engage with both reality shows in detail, looking at how various characters have been treated, and how they have portrayed themselves. Then, looking at how and when the liberalization of India happened and affected changes, I use Rosalind Gill's (2007) understanding of postfeminism as a starting point—as a sensibility instead of a total shift or a historic moment. This framework is useful because the sensibility “emphasizes the contradictory nature of postfeminist discourses and the entanglement of both feminist and anti-feminist themes within them” (2007, 149). Departing from this starting point, I then articulate my findings with the works of several theorists, including Angela McRobbie, Chandra Mohanty, Jesse Butler, and Simidele Dosekun to better situate the rise of postfeminism in the Indian context.^3^ I conclude with a critique of both the shows.

## Arranging love in the Indian context

In the Indian subcontinent, the term “love marriage” —a union based on romance—is often juxtaposed with the term “arranged marriage,” where kinship structures and caste endogamy play parts in determining who marries whom. While marriages of both types coexist in the region, Western media discourses and popular imaginations tend to overrepresent the notion of the “universal” arranged marriage (Pande, 2014), meaning an arrangement where neither the bride nor the groom can exercise much or any agency, and the families set up the union as an alliance between the families.

A love marriage, the romantic liaising between two people, where it seems to be all about individuals connecting to each other is seen to be a westernized ideal in India that either people aspire to or else it is looked down upon by many as people who are disrespectful of their culture. This disrespect is not just accorded to people who marry without the consent of their families, but also to people who marry outside their caste, and outside their religion. Both these transgressions have been the cause of violence in India—with honor killings, revenge killings and this emerging idea of Love Jihad leading the way (Sharma, 2020). Indeed, much of commercial Indian cinema thrives on these tropes (Jha, 2018; Ezhilarasan, 2020; Nathan and Ramnath, 2021).

In 2005, the Indian Human Development Survey (IHDS)^4^ (Desai et al., 2018) collected data to explore the spread and extent of mixed marriages in India (inter-caste, inter-class, and inter-religious). While it was noticed that there was a general growth in percentage for both interfaith and inter-caste marriages, in real numbers it is far lower than the number of endogamous marriages.

According to the Survey, the primary reasons attributed to mixed marriages are socio-economic status, bargaining power for women from better educated backgrounds, financial affluence. Also, there is an upward swing in the age-group of the women surveyed. Such mixed marriages are also urban-centric and were seen more among urban Christians than any other religious community. Both mixed marriages and Inter-caste marriages seemed to go up with more education.

The IHDS report also extrapolated that those Indian states that are considered to be more traditional had the lowest number of inter-faith marriages. Traditionality in India dictates that a marriage goes beyond two individuals. In the more traditional states, the parents deciding the spousal selection is highly common. The impact of westernized and western education and economic upward mobility has influenced the numbers of mixed marriages. It also acts as a critical measure of socio-economic upliftment.

India is fraught with different caste and religious identities, and a sense of superiority and “otherness” prevails among them. Thus, despite economic upliftment and educational exposure, caste and religious endogamy carries on undeterred, as seen in the report. There is a greater possibility of an inter-class marriage within the same caste and same religion due to upward mobility and higher education.

All this said, arranged marriages, of course, are not unique to the subcontinent. However, the way families get together to arrange the matches of two “suitable” people, and the industry it perpetuates is probably unmatched, as the wedding industry in India is a multi-billion-dollar industry. In India, as well as in Indian-origin communities across the world, arranged marriages are not limited to a particular religious, class, or caste group. A misplaced pride in the culture of families coming together, rather than individuals, often places people looking at getting into relationships under undue pressure.

The more traditional and conservative a community, higher the chance of people being persuaded to meet the local matchmaker. Because of the caste system still being prevalent among Indians everywhere (particularly Hindus), many people are expected to marry within their caste. In fact, all Indian newspapers carry matrimonial advertisements that are segregated along caste lines, religious lines and sects and sub-sects, depending on the region, as do the matrimonial websites.

The wedding industry's survival depends on a hegemonic structure that perpetuates gender stereotypes (given that same sex/same gender marriages are still neither a local nor a pan- global legal reality). In many parts of the world, there is a ceremonial giving away of the bride. Treating women as property, cattle, chattel is often made into a spiritual and emotional issue through rites and rituals, especially in Hinduism. Also, the practice of dowry/bride price/*mehr/lebola* (depending on the community) makes it a large financial transaction.

*Indian Matchmaking* catapulted the notion of arranged marriages into the drawing rooms of the Netflix watching audience. It became a highly talked about television show across the Anglophone world. Apparently on the opposite side of the spectrum is *What the Love!* is a show where Karan Johar—a renowned Bollywood filmmaker—takes it on himself to take a few individuals on a journey to find love. It is much like the shows that have been the makings of daytime television in many countries of the global north (for instance, McRobbie, 2004). What is ironic are the inadvertent similarities between the two shows in their treatment of people, and reifying stereotypes in femininities and masculinities.

## Indian matchmaking

Many Netflix viewers had only heard about South Asian (read Indian) arranged marriages. *Indian Matchmaking* gave the non-South Asian viewer a glimpse into the first step of arranged marriages within Indian families both in India and abroad—that of the matchmaker. The potential brides and grooms on the show all came from middle to upper-middle class financial backgrounds, primarily Hindus—from lawyers to entrepreneurs to diamond merchants to high school counselors, it covered quite a wide spectrum. There is a certain farcicalness about the entire show. It seems to be more of a conversation starter about norms and traditions vs. an in-depth understanding of the custom of arranged marriages in India.

The show concentrates on middle and upper middle-class Indians in India and the United States. The matchmaker is a woman called Sima Taparia, who introduces herself to all her clients as “Sima from Mumbai.” Sima's own adult life almost started with her own marriage—at the age of 19 and a half, in 1983. For her, it was inconceivable that she would go into a love marriage (Episode 2). She puts a lot of focus on the “biodatas” (a term used widely in India instead of résumé) of the candidates. “Everything we come to know by the biodata” (Episode 1).

Sima has a very particular take on marriage—she is a mix of superstitions and traditionality and believes that matches cannot happen without families being involved. At one point, she says that “matching horoscopes is like insurance in a marriage… even if horoscopes don't match, many people proceed, especially in the US” (Episode 5). There is a strong sense of trying to normalize arranged marriages through the show, and how successful they are. Many of the episodes open with vignettes from couples who have been together for many years. One couple, Suresh and Sunita Kabra, say that they have been together for 42 years, and they hadn't met till the day of their wedding (Episode 5). Another couple, Raj and Rashmi, met several years back through a newspaper, India Abroad, and they matched through the matrimonial columns. Rashmi jokes about being a “mail-order bride” (Episode 6).

The show operates transnationally between the Indians in India and the diaspora in the US. The potential candidates who have allowed themselves (and their closets, in some cases) to be scrutinized by Sima and the audience are Aparna Shewakramani, a 34-year-old attorney from Houston, Texas, Pradhyuman Maloo, a 30-year-old jeweler from Mumbai, Nadia Christina Jagessar, a 33-year-old event planner from New Jersey, Vyasar Ganesan, a 30-year old teacher and college counselor from Austin, Akshay Jakhete, a 25-year-old businessman from Mumbai, Ankita Bansal, a 30-year old businesswoman from New Delhi, and lastly, Rupam, a 36-year-old Sikh woman divorceé from Denver, who has a daughter from a previous marriage. Most of these people have tried dating and having relationships without any success. Hence, they decided to fall back on the cultural “advantage” of being Indian (or of Indian origin) and going through a matchmaker.

Throughout the show, what becomes evident throughout the show is that some people have strangely high expectations from this matchmaking process. Although dating apps' algorithms have not worked for them, they expect a human being to anticipate all their shortcomings and flaws and get a perfect match for them. Sima does everything from going to face-readers to astrologers to see if her candidates are well-matched for each other. However, we find out after the show that not even one of her matches actually worked out.

The candidates come from very varied personal backgrounds. Aparna is quite fixed on what she wants, but despite the trials of matchmaking, she feels that she is still in the need for a matchmaker because what she is doing by herself is not working. Pradhyuman feels he is quite a catch and keeps rejecting proposals because he wants to find someone he feels attracted to immediately *via* their photographs. However, he is forced to confront his loneliness when among his married friends. Nadia, despite being very warm and open-minded, often gets rejected by Indian people because of her not being perceived as Indian enough. Vyasar, who is generally very easygoing, has what Sima calls a “complicated family history.”

Akshay was educated in the US. However, despite this exposure, he seems to be quite tied to his mother's apron strings. He was surprised that he had to do his own laundry when living abroad. He barely has any say on his marriage—his mother actually makes it a point to mention this over dinner 1 day—“you get married this year [your brother and his wife], have a baby next year.”

Ankita Bansal, a Delhi-based entrepreneur, is often called “*chalu*” (a Hindi word that means characterless or immoral). Rupam, with her divorce and her child from a previous marriage, makes her a difficult candidate to match—“Divorce carries stigma, especially with a kid.” Sima tells her, “You will get less options” and “you will have to compromise.” We also learn through the episodes that both Aparna's and Vyasar's parents are divorced, and that Vyasar's father is an ex-convict who was convicted for attempted murder.

With Aparna being an attorney, Sima shares with the audience that “if the females are lawyers in India, people are scared.” Aparna's criteria for meeting potential matches, among many points, is primarily that the person must be a US citizen and that he be of North Indian descent as she herself is Sindhi.^5^ However, Sima initially brushes aside the criteria laid out by Aparna, saying “many of these things are not important for a happy married life”—a relationship cannot be tailormade.

Sima also feels that “Aparna is the hardest type of candidate to match because she thinks finding a life partner is like ordering from a menu” and that “Aparna has to compromise” (Episode 1). When Aparna puts down an age criterion after a failed match Sima says that she is “not stable” (Episode 2). Yet, when we meet Pradhyuman, the Mumbai-based jeweler, we learn that he has rejected over a 150 proposals over a period of 18 months based on people's looks in their bio data photographs, and Sima doesn't have anything negative to say about him (Episode 1).

Sima shares with the audience that Pradhyuman “has very high expectations,” that he “has to change his superficial nature,” but the boy is “good.” This “good” boy ignores several matches that Sima sends his way, and finally meets one of the matches, Snehal, a girl from Jalgaon. While they seemed to get along, from the very beginning of the meeting, Pradhyuman is dismissive of Snehal for being from a small town. He calls her “simple” and “homely^6^” (Episode 3).

The show opens with vignettes of people who say things like “the girl has to be a bit flexible,” and different aspects of arranged marriages are brought to the forefront—caste, height, “slim, tall, beautiful, but with a good nature,” and the importance of astrology is brought up. While the Indian constitution has abolished casteism, and discrimination on the basis of caste, it carries on unchecked throughout the Indian subcontinent and the diaspora.

What is also interesting is the euphemisms that people use when they don't want to refer to caste—similarly situated, similar cultural backgrounds, etc. While to the untrained eye, it might mean that a person who's Indian wants another Indian person to have more cultural connect, in actuality, there is pressure on marrying within the same community, the same strata and so on. It must be mentioned here that Sima does set people up with others who are from different regions in India, which means there may be cultural differences galore.

When Nadia's family discusses her Indian-Guyanese heritage, she mentions that, often, for other Indians, she is just not Indian enough—they date her but marry someone who is more Indian than her. She still prefers Indian men because she feels there is a larger cultural connect there. When Sima presents her with matches, Nadia's family asks Sima if the prospective grooms are aware of their family heritage, and her prompt reply is that “caste” is not a problem with anybody.

This aspect of Nadia's Guyanese heritage is particularly pertinent here because many people of Indian origin who hail from various African countries, or the Caribbean are descendants of many indentured laborers who were taken across by the British in order to get cheap labor for their plantations. This has a particular caste aspect associated with it as well, and it often creates a sense of superiority among other Indian diaspora who may have chosen to move to various countries out of choice. Sima says that she is a “a good girl, but match is difficult,” probably because of this very reason (Episode 1).

Being a “good girl” but difficult to match with prospective suitors is something that comes up with Rupam as well. However, when Sima does get her two prospective matches, her father rejects one of them on the basis of the fact that he was married to an “American” (read as white) woman previously (Episode 7).

For Ankita, Sima decides to team up with a fellow matchmaker, Geeta, who initially comes across as someone quite different from Sima. While Sima focuses more on bringing two families together, apparently Geeta focuses more on why the candidate wants to get married. While this seemed like a promising start, when Geeta meets Ankita to find out what she wants, she says that “it is our duty as a woman to understand that, in a marriage, the woman gives the emotional side of herself much more than the man does.” Ankita shares with the audience that Geeta made women feel “like inferior objects.” Despite this, she sets Ankita up on a date with Kshitij, who she gets along with quite well. Despite Ankita's misgivings about Geeta, she feels understood about the type of partner she wants (Episode 6).

Geeta had held back a vital piece of information—about Kshitij being a divorcee. On getting to know this, Sima makes light of it, despite her own reservations on Rupam's divorce. Also, the fact that it was hidden from Ankita is brushed aside by Sima (Episode 7). Sima recommends Ankita to the counselor, Varkha, where Ankita confronts many of her body image issues. However, her connecting with Varkha, the guidance counselor/life coach, also made her realize that she wanted to concentrate more on her entrepreneurial skills rather than running behind getting married (Episode 8).

Despite the double standards that we see in Sima's treatment of her male and female candidates, the show ends with a lot of possibilities for the future. Akshay gets engaged to Radhika in Udaipur, with his mother beaming with pride. Rupam finds someone a dating app, and Sima is really happy about the fact that she found someone. Yet, as mentioned earlier, nothing actually panned out.

## What the love!

*What the love!* is a distinct departure from *Indian Matchmaking*, but as I show later on, there is a striking similarity between the two shows. In *What the love!*, Karan Johar plays the role of matchmaker and takes six candidates on a quick makeover journey [both emotional and physical, somewhat like the old *Queer Eye for the Straight Guy* (Collins and Williams, 2003) rebooted later as the *Queer Eye* (Collins, 2018)] while Johar tries to talk them down from the romantic ideals that his films have set for many people across the subcontinent and the Indian diaspora. The six candidates are chosen based on how the hosts feel they can be fixed or elevated from their current situation.

The first episode is a party, albeit a bit contrived, where several people ostensibly looking for life partners have been gathered. Karan Johar, the host of the party, shares with the audience that the party is full of people who are “fashion disasters on display” who need a lot of help. For helping him on his savior journey, he is joined by stylist Maneka Harisinghani and make-up, hair, and grooming expert Shaan Muttathil.

The three co-hosts chat to the people they want to select, and touch upon many issues that affect people in Indian society. Issues that come up are the societal expectations of women needing to get married in their twenties, weight, the exhibitions put on for prospective suitors, the general assumption that people are heterosexual, and much more.

Beyond the one-on-one chats, the party also has two games, one being rolling the dice, and the other being spinning a wheel. These games allow Karan Johar to ask questions to random people, but also for him to share something about himself. Johar talks about having paid for sex with a man—something that is a reality that we all know exists, but seldom does anyone talk about it.

At various points of the episode, it seems that the party guests are keen on making the most of their screen time, and they carry out conversations that wouldn't have been out of place in one of Johar's romantic cinematic productions. Through all this, Johar selects the candidates, both men and women, who need “help,” for their show—mostly straight, and one gay man: Aashi, a pathologist, who's thrust into the marriage market by her parents, where she is severely judged; Rabanne, a gay male model and graphics designer, who feels it is time for him to look more seriously into relationships; Geetika, a fashion designer, who survived a near-fatal accident that required her to undergo multiple stitches on her face, leaving her with a scar, and many insecurities; Rina, who's boyfriend of 2 years told her to her face that she isn't pretty and ghosted her,; Vaibhav, who has spent most of his adult life trying to build his career, and hiding behind his former-fat-boy image; Rameez, who seems to know what he wants but his hyper-planning is all about masking insecurities.

Certain aspects of the first episode allowed us a glimpse of what was to be expected. The first person they talk to, Aashi, mentions her caste almost immediately. While this wasn't a scripted show, some critics felt it could have been edited out (Shukla, 2020). However, given that the reality of the Indian marriage and love market is defined by these boundaries, it was a pertinent aspect to leave in. Secondly, during the games, Johar makes it a point to speak about equality for women, and women's empowerment, but it came across as more of a jibe against a person who said that men should pay on a date than a meaningful foray into understanding the mindsets of women within patriarchal and postfeminist constructions.

What the first episode doesn't tell us is that the overall makeover is all done within a day—emotional, mental, and physical—and that the specialists brought over to help with them (except the physical makeover aspect) are celebrities who may have faced similar issues in their lives without necessarily having the expertise to deal with the issues presented to them.

Each episode starts with Johar, the host, chatting with the candidates before sending them off with a celebrity on a mock date that he observes and comments on. After that, the candidate is sent for her/his emotional/mental makeover, and lastly for a physical makeover—clothing, makeup, hairstyle. Then Johar sends the candidates on two actual dates (followed by a camera crew), after which the candidate must decide who she/he would like to meet again. The show's cliffhanger moment is whether the date chosen has also chosen the candidate. The dates, for the most part, are stereotypically romantic, and somewhat like Johar's film sequences.

The second episode starts with Aashi, the pathologist. She brings up her issues with size, her rejection in the marriage market−11 times in a row, her lowering of expectations from what she wants in a life partner, her sexual abuse at the hands of a relative as a child, and so on. She also mentions how she was made to stand on a weighing scale by a prospective groom's mother, and how they were astounded by the fact that she wasn't over 90 kilos in weight. Her caste aspect is no longer referred to. After this initial chat with Johar, she is sent on a mock date with an actor, Arjun Kapoor, a mental makeover by comedienne Mallika Dua, and then a physical makeover by the stylist, Maneka, and Shaan, and the grooming expert. Maneka wants to “jazz” her up and make her look “foxy.” After her makeover, she is sent on two dates—one with a doctor, and another with a transportation entrepreneur.

While the show opens with the promise of bringing people up to their fullest potential, what it does is fall within the tropes that it had seemed to make promises against. It might be unfair to put the entire blame on the show. The candidates themselves seemed to be keen on fitting into existing tropes around masculinity and femininity, and certain aspects of heteronormativity. For instance, Rabanne, the sole gay candidate, makes no bones about wanting to be the “damsel” in a relationship, which comes dangerously close to subscribing heteronormative roles and patterns in gay relationships.^7^ He even declares that he wants to be carried around (physically).

When Rabanne is sent on two dates, one with the epitome of masculinity and machismo, Karanbeer, he feels a bit too overpowered and pushed with the physical advances. On his second date, he meets with a makeup artist, Aadarsh, who he almost immediately asks him about his sexual position preference -whether he's a top, a bottom or versatile^8^—which takes Aadarsh aback a bit. While Aadarsh does not fit the typical notions of masculinity, it may be safe to assume that Rabanne did not ask Karanbeer the same questions because in many instances, aggressive masculinity is equated to being a top, and Aadarsh's general demeanor being typically masculine may have elicited the question from Rabanne's side.

The two heterosexual men who were brought on the show for makeovers, Vaibhav and Rameez, seem to be quite the opposites of each other. Vaibhav has concentrated on his career to a point where it has become synonymous with his identity, and Rameez is a former flight attendant whose claim to fame is that he has been to over 75 countries, but strangely, is poor in geography. When Vaibhav, who is somewhat shy and retiring, is sent on a prep date with Sunny Leone, a Bollywood actor and former porn actor, she is of the opinion that there is nothing better than a man who takes control, that “he's a boy, he needs to become a man.” While she speaks of how Indian mothers mollycoddle their sons, and that in turn becomes emotional baggage (something that we see in *Indian Matchmaking*), Leone's idea of masculinity is borderline toxic.

Rameez, on the other hand, seems to have very fixed ideas of what he wants in life, with a laundry list of things that he wants from his partner. When he tells people that he has been to over 75 countries, his being a former flight attendant begs us to surmise that he has been there on work rather than to explore. He seems to want to give the impression of being more worldly wise than he actually is. He comes across as someone who has been pampered a lot, but is more shy than arrogant, and rather confused. He seems to have a very specific notion of masculinity and relationships, and for him, a relationship seems to be more about ticking boxes rather than actually experiencing a relationship. When he meets with Cyrus Sahukar, a Bollywood actor and former video jockey, for his mental makeover, Sahukar points out to him that it seems like he has a wedding dress ready, and whoever doesn't fit into it is unsuitable. Johar, too, pointed out that he seems to want a woman to blend herself into his life if only to tick his boxes.

Two more women come on the show—Geetika, a fashion designer, and Reena Kumari, who works in a corporate. Geetika went through a life-altering incident, where she was in a road accident and her face was severely affected and left behind a scar which makes her extremely conscious. Reena left behind a toxic relationship where her partner ghosted her after 2 years, and that too on their anniversary. When she is on a prep date with Bollywood actor Saif Ali Khan, she is taken through various scenarios by Khan, and it turns out that she has a pattern for allowing people to take control of her. Even when she was sent for two dates, she was instantly attracted to the person who came across as more typically masculine and had the air of being more in control.

Geetika's car accident happened on her 21st birthday. Since then, she has always felt as if people are curious to know more about her facial scar as opposed to her as a person. While the scar was unnoticeable till it was pointed out, her focusing on it, despite being conventionally pretty, just goes to prove the point that the stereotypes of beauty that are fed into people, and how they are expected to portray themselves.

### Of weddings, neoliberalism and postfeminism in the desi context

*Indian Matchmaking* and *What the Love!* are entertaining, and many of the characters are quite endearing as well. They have been the source of much enjoyment, cringe-fests, and a ton of discussions, both academic and non-academic. As dissimilar as Johar and Sima might seem, they are branches of the same tree. They both are acting as matchmakers—one traditional, one modern. In both shows, they, advertently or inadvertently, pointed out what the “faults” were with the people who were up for being matched. In one, there was giving into superstition, in another, in good western makeover show fashion, they were given life-skill advice by special guests. Yet, why shouldn't they be there? After all, reality shows such as these have been a part of day-time television internationally for a long, long time.

However, these shows bringing together Indians from across borders becoming a part of an international platform is because they are a manifestation of a much larger movement, both a part of the Bollywood culture industry, as we shall see below. Also, just the way they are part of something much bigger than small-screen entertainment, the discussion that has been brought about goes way beyond the shows.

India's (economic) liberalization came hand in hand with global neo-liberalization, at the end of the twentieth century. Various corporates wanted to be the vanguard of this movement. To signify the shift, the Miss World Pageant hosted in Bangalore (now known as Bengaluru) in 1996 was the ultimate “we are here” by the Indian corporate world and more. However, there were protests galore—protests from both the right wing as well as the left wing. One segment saying that it went against traditional Indian values (the ideal Indian woman) and the other as to how it was nothing but a display of capitalism and commodification.

The organizers saw it as a mode of going global, and as the perfect steppingstone onto the development bandwagon. Thus, this pageant becomes foundational for a rise in consumerist passions and aspirational behavior, toward getting into the modeling/beauty industry. Yet, at the same time, it became a site of contestation of values clashing on all sides. While not new, the pageant can also be seen as a catalyst for further enforcing the usage of usage femininity as strength, and consumerist behavior as a sign of success and social acknowledgment (Mazzarella, 2015).

The neo-liberalization, along with the movement toward a global village, contrasted with rising rightwing ideology and nationalism is creating new uncertainties for women to navigate these realms (Mitra-Kahn, 2012). These uncertainties have also fed the need for people to showcase what they are capable of financially—or at least show, even if they don't have the capacity. This is where the intersection of neoliberalism with postfeminism is all too present in Indian society transnationally. Women supposedly make life-defining choices out of their own free will but end up being acknowledged only by their consumerism. The uniqueness that they are apparently seeking through their consumerist choices is actually being driven by the market paradigm where they are all trying to fit into what is seen as appropriate. Gill (2007) speaks of postfeminism as sensibilities in which “the notions of autonomy, choice and self-improvement sit side-by-side with surveillance, discipline, and the vilification of those who make the ‘wrong' choices (become too fat, too thin, or have the audacity or bad judgment to grow older)” (2007, 163).

The neoliberal turn that India made fed into what can be eponymously termed as the Bollywood Wedding,^9^ “a class-based, gendered response to India's turn to neoliberalism” (Kapur, 2014, 93). The big Bollywood wedding is born out of the notions of tradition, familial bonds and a glossy version of feel-good nationalism showcased in Bollywood cinema as well as several soaps across the multiple television channels in neo-liberal India. Weddings in India have always been socio-culturally meaningful along with a showcasing of economic might. Yet, with the new age, weddings have become sites of high levels of conspicuous consumption and materialism. The consumption is a head-nod to having arrived in society. Indeed, the same Bollywood culture that is promoted both by private individuals and the public body is all too present in the way Indian-ness is promoted outside India. With all the glamor and glitter of Bollywood, it becomes a reinvention of the same tropes that perhaps generations of people have tried to undo in post-independence India (Kapur, 2014).

The wedding scenario, particularly Hindu weddings, are shown as a celebration of two families coming together. Archaic rituals such as seeing the bride for the first time, celebrating the son-in-law, offering dowry by the bride's father (only to be refused by the groom's family), giving away of the bride, and caste endogamy are showcased *ad nauseum*. This is where there is a break between diasporic cinema and proper Bollywood productions. Diasporic cinema, such as *Monsoon Wedding* and *Bride and Prejudice* (Chaddha, 2004), is seen to exist outside the mainstream cinema productions, “independent or interstitial because of their supposed marginalized mode of production within the context of xenophobia, empire, nationalisms, and global capitalism” (Desai, 2013, 207).

Yet, for some, there is a conflation of diasporic productions within Bollywood. Movies like *Monsoon Wedding, Bride and Prejudice* are set in India, which helps it remain cloaked in Bollywood-ness, but meant for an external audience. Similarly, the reality shows spoken about in this paper are structured for a larger audience, beyond the subcontinent, beyond the diaspora. The platform lends itself to a global audience, not just relating to the North or the South (Desai, 2013).

Making marriages the site of conspicuous consumption, replicating archaic rituals, deconstructing feminist stances, etc. is a bastion of the middle class. Indeed, the middle class is particularly vicious in the way it practices gender discrimination—especially with poor people. The new middle class as a political construct in an amalgamation of being “a demographic category, a potential market, or an identity associated with consumerist lifestyles—quiet a shift from being the site of anti-colonial struggle. At the same time, it is juxtaposed with being the primary bastion of liberalism, being anti-caste, secular, despite having strong affiliations with the Hindu right (Bhatt et al., 2010).

Today, the ubiquitous Indian wedding is the posterchild of “an unabashed departure from an earlier Gandhian-Nehruvian embarrassment around conspicuous consumption in a predominantly poor nation” (Kapur, 2014, 98, 99). It is also a landscape of enacting and redesigning traditionality, of how people ought to conduct themselves within the confines of a culture. This is where several aspects of postfeminism come in. Some specific aspects of postfeminism that remain constant are: “femininity is a bodily property; the shift from objectification to subjectification; the emphasis on self-surveillance, monitoring, and discipline; a focus upon individualism, choice, and empowerment; the dominance of the makeover paradigm; a resurgence in ideas of natural sexual difference; a marked sexualization of culture, and an emphasis upon consumerism and the commodification of difference” (Gill, 2007, 149). The wedding is also a scene of portraying uniqueness while giving into hetero-patriarchic tradition.

The postfeminist woman's perceived empowerment is through acts of feminization. She is required to absorb various feminist ideals and simultaneously move themselves away from the political sphere. It rides on the assumption that the goals of the feminist movement have been achieved, thus ostensibly (un)doing feminism (McRobbie, 2009) into a more individualized narrative. This notion of “femininity as a bodily property” is to be used and weaponize it (Gill, 2007, 149). While within this framework, women are to make their own choices, it feeds into the trope of what is considered generically “womanly” and sexy. While some see these specific acts as daily acts of empowerment and political choices, others, particularly traditional feminists, view it as moving away from collective, political action toward individual consumerism.

This goes hand in hand with the idea of “self-surveillance, monitoring, and discipline” (Gill, 2007), where women are expected to constantly school and align themselves with socio-cultural expectation. Oddly, this self-surveillance is seen to be a personal choice, and not something foisted on them. While this is not new, it has reached a new level of being extended into one's intimate spheres (155).

Media, contemporary culture and even the State has used feminism to signal the emancipation of women, to a point where gender equality (whether it is a ground level reality or not) is understood as common sense. Various successes of feminism are used to showcase why feminism has become irrelevant in today's era (McRobbie, 2009; Roy, 2012).

Postfeminism does not discount the existence of feminism. Instead, it posits itself as a replacement of feminism with an understanding that the previous battles of feminism are displaced by the postfeminist ideals of individualism, choice, and empowerment. Particularly, with the rise of neoliberalism and capitalism, as the neoliberal movements absorbed the left's discourse selectively, McRobbie argues that neoliberal capitalism has actively attacked and undermined feminism and feminist movements (2009).

Postfeminism works to conceal new modes of gender regulation and relies heavily on a “framework of capacity, freedom, change, and gender equality” (McRobbie, 2009, 51). The “new sexual contract” moves away from the previous limiting gender regimes and works through “incitements and enticements” toward “both progressive but also consummately and reassuringly feminine” but the “abandonment of critique of patriarchy is a requirement of the new sexual contract” (57).

However, McRobbie falls short of including women of color within her understanding of postfeminism, as something that reinforces racial divisions and reinstates whiteness as the racial standard. Butler (2013) challenges this assumption—is postfeminism for white girls only? The assumption that postfeminism excludes women of color, or that they do not appear in postfeminist pop culture, per Butler, seems to not consider the several women of color who are enacting postfeminism. With the number of Black and Latinx popstars and girl groups themselves, it is evident that there is a representation of postfeminism among women of color, who “clearly embody and enact postfeminism: they embrace femininity and the consumption of feminine goods; they espouse a vocabulary of independence, choice, empowerment, and sexual freedom; and they construct themselves (or are constructed by others) as heterosexual subjects” (48).

Thus, Butler wants an “Intersectional Approach to PostFeminism.” It is certain that the representation of women of color in media is according to the standards of the heteronormative white woman of a particular class and a stereotype—as long as the women of color fit into the “normative conceptions of race, class, gender, and sexuality” (2013, 49, 50). These women bring diversity into the media constructions for sure but are portrayed only in a particular light—the torchbearers of the exotic, the sultry (and in many ways, the primal) other (50, 51). Taking off from Butler, Dosekun (2015) feels that postfeminism itself is considered to be “Western,” and the sensibility has been “deemed as ‘white and middle class by default [because], anchored in consumption as a strategy (and leisure as a site) for the production of the self”' (Tasker and Negra, 2007, 2 in Dosekun, 2015, 961).

Hence, while the inclusion of women of color may be seen as an unsettling force within the otherwise white postfeminism, it comes at a cost. Dosekun expands the interrogation of feminism not just through an intersectional approach, but also through a transnational approach, “to designate that which exceeds and traverses such boundaries, as well as the analytic mode of thinking across them” (961). She questions the solidity of the constructions of the West/East (and in turn, Global North/Global South) divisions, and the limitations posted by scholars which has prevented a better engagement with postfeminism outside the west.

Much earlier, Chandra Mohanty had raised a similar critique of western scholars looking at the global south through a singular lens. Her critique of western scholars constructing the “‘Third World Woman' as a singular monolithic subject” (1988, 61) has aided in problematizing and evaluating the West/East, North/South narrative. Within this narrative, the West is seen as a site of progress, espousing the causes of postfeminism, and the East as a site of victimhood of women where feminism is yet to perform its magic to emancipate the oppressed (Dosekun, 2015, 962). This notion of “us” vs. “them” is similar to the white savior trope (Mutua, 2001) that has often been seen in different colonial and post-colonial narrative constructions. What is often ignored is that women from the global south live in plurality. Thus, treating them as a monolith is reductive to say the least (Mohanty, 2003). With the plurality comes the performance of local feminisms, which are also ignored.

Dosekun questions McRobbie's concept of the non-western “global girl” who is a “*tame, derivative copy of its putative Western original*” (2015, 963), who aspires toward a level of consumerism that helps her toward the goal of becoming the desirable Westernized woman. This “global girl,” while aiding in creating at least one aspect of the subject, comes dangerously close to the monolithic third world woman (Mohanty, 1988). It conflates women of all classes and different levels of privilege and precarity together, which disallows an understanding of how postfeminism reaches particular classes of people, and how they perform it without necessarily trying to aspire to be western (Dosekun, 2015, 963).

By looking at class as a central criterion for inclusion in the said “post-feminist global sisterhood,” Dosekun moves away from others (964), and suggests that we consider postfeminism from a transnational perspective. Doing this, Dosekun offers us a framework within which post-feminism fertilizes the imaginations of the “feminine subjects” who are able on all levels to buy into what postfeminism has to offer (966).

What Dosekun suggests in the global south context, given the tremendous inequalities, grassroot level feminism works parallel to middle-class women empowerment where feminism is performed by constructing the working professional woman and is further commodified through the consumption of the beauty and fashion industry. With the fact that grassroot level feminism, especially in a place like India, is more about ascertaining access to civil and political rights for women, higher class, educated, urban women possibly bypass feminism directly toward consumption-based postfeminism (967). The undoing of feminism by post-feminism is a transnational phenomenon—for those women who believe that they are empowered, they have it all, and they are exercising their choice through consumption, and they have little idea as to how they came to have it all.

As an example, Dosekun refers to Parameswaran's (2004) where she speaks of how global beauty pageants are producing beauty queens for India who are the new perfect example of performing an empowered Indian femininity which is, unsurprisingly, unavailable for most Indian women. This particular representation not only satisfies the middle-class consumer but also the nationalist sentiment of how India has become truly globalized (2015, 969). It also pushes forward a highly feminized femininity, that is also in many ways not just sexualized but heterosexualized.

Donner's (2013) work in India highlights how different feminisms and status are intertwined. Lower middle-class women do not consider going out for work in order to keep their status separate from the people they are socially closest to—the lower working class, often working as domestic help. The “us” and “them” are fenced off in this way. On the other hand, Donner's interactions with more westernized families had several women of the household working. This is a true juxtaposition of becoming part of the labor force for survival vs. emancipation. This goes parallel to the juxtaposition of uneven new developments with the old customs/traditions.

Similarly, Grover's (2011) work in the Delhi slums is very telling—she explored how lower caste men and women marry and remarry with far fewer restrictions visa vis their upper-caste counterparts (within the urban poor). Within Hinduism, there is a general lack of the acknowledgment of divorce. Although legally there are steps that can be taken, it is stigmatized to a great extent. This reflects within the diaspora as well. Grover laments how the Indian middle-class has become the self-appointed representative of global trends. The notion of divorce is studied mostly within the context of this middle class. However, her work in the Delhi slums is telling—about how the “husband” needs to be a good provider—with often this ideology that he should be the sole bread-earner. It is a bit of a fall from grace if the wife has to go out to work. Also, a failure to provide for the home might induce a woman to look for another person as a protector and provider. The moralities affiliated here are around being hard-pressed, the need for protection, etc.

There is no one-size-fits-all when it comes to exercising agency and feminism in a place like India, where different aspects of caste, class and religion constantly intersect. Having parallel feminisms has led to different stances when it comes to some women who have entered a formerly all-men's arena in India. Yet, despite this, many women do not like being termed as feminists. For instance, in Indian cinema, several women directors who have made ground-breaking cinema, showcasing the rage and the taking back of control and agency of the oppressed, the marginalized, the disenfranchised, do not like aligning themselves with the word “feminist.” There is a tendency of thinking of the word “feminist” as ugly and loaded. When Bengali Actor-Director Aparna Sen was interviewed, she refused to be called a feminist despite having contributed to feminism with her work (Dutta, 2019, 16). Reema Kagti, a Bollywood film maker, has made films with strong women protagonists. She seems to believe that Bollywood has come of age where one doesn't need to differentiate between the genders of the directors. However, at the same time she has been called out for being too “aggressive” with behavior that is happily accepted of male directors (Dutta, 2019, 119, 133).

## The aftermath—Matchmaker, matchmaker, make me a match!

Derné (2003) studied how a decade of globalization from the early 1990s created a burgeoning upwardly mobile consumerist middle class in India that tried to situate itself as global citizens while thwarting any influence of it on internal, traditional setups. He speaks of an Indian man, Amit, who watches everything that global media has to offer, who wants an arranged marriage, but fears that the new global media that was available for everyone's consumption was “distorting the desires of the younger generation” (12). Derné points out middle-class Indian men like Amit were absorbing only what fits into his socio-cultural understandings—valorization of masculinity in the form of violence because it feeds the local ideals of patriarchy but ignoring the challenges to traditionality.

Two decades later, we see the same replicating itself in both shows—with Sima in *Indian Matchmaking* saying that Aparna is too educated, and that in India, people are afraid of women lawyers, and with Aashi, the pathologist in *What the Love!* recounting the number of times she was made to parade in front of prospective grooms, and a time when she was actually made to stand on a weighing scale by a prospective groom's mother.

*What the Love!*, while trying to break boundaries and bringing to the forefront multiple issues that plague Indian society, doesn't move away from stereotypes where women are made to seem more feminine and fragile, men are made to seem more masculine and macho. Even when women are portrayed as powerful, it is within the constructs of what is deemed powerful by normative standards.

Makeover shows that give people makeovers to not become more presentable for a career, but to fit ideals for a relationship reifies these standards. For instance, Aashi, during her makeover, was made to look “foxy,” as mentioned earlier. That is easily interpreted as giving in to heteronormative standards on what is sexy. While the physical makeover might be a boost for Aashi, who has been through the wringer with people judging her on her looks and weight, it cannot help but reinforce toxic social standards. The choice of words is extremely important here. Johar, while trying to set people up on prospective romantic adventures, tries to bring together way too many aspects of social reality than it can handle and lands up fitting into conventionalities that it initially seemed to want to walk away from.

While the candidates on *What the Love!* were not followed up on, the cast from *Indian Matchmaking* have been interviewed on different platforms. One of the first programs was an online interview with (Netflix India, 2020). She chats with all the contestants and found out that Akshay broke off the engagement in record time—what happened to the poor fiancée, we don't get to know. Also, Aparna says that she received a lot of support from women everywhere about her not giving into pressure to get married and being herself. Ankita said that she thought Sima had made a difference to her life by introducing her to a life coach and had made her more focused on her career. Almost everyone who was interviewed by Singh mentioned that they had people calling them up for re “*Sisterhood with Shaili*,” a program hosted by Shaili Chopra on *SheThePeople TV*, interviewed some of the candidates. That is where Ankita said that “the word ‘compromise' is very frivolously used in terms of marriage. Marriage, in itself, is quite an anti-woman construct. In India, especially in Hindu customs, the daughter is literally ‘donated' at the wedding by the father, and the more anglophone western version has the giving away of the bride by the father. Irrespective of how they reach the altar, the mode remains the same. Bridehood turns out to be a parade of beauty standards and wifehood turns out to be about learning adjustment and compromise” (SheThePeople, 2020).

With *Indian Matchmaking*, Netflix normalizes and even dignifies traditional matchmaking, whether it is a matter of social pressure that makes the candidates come forth or whether it is just general loneliness. Some people who have broken away from typical marriages do not necessarily stay away from marriage, but rather, they marry on their own terms and not the terms that society dictates. Society validates marriage and married women. Unmarried women stand out because wifehood and motherhood are seen as the essence of womanhood.

As mentioned earlier, *Indian Matchmaking* opens with these vignettes of older people who got married through different sorts of arrangements—from advertisements to matchmakers. They present a picture of heteronormative stability, dependability, and contentment. It feeds into the ethos of the show, as to why arranged marriages might be better after all.

Yet, while not saying a single word about it, the vignettes speak volumes about caste and religious endogamy. The Indian landscape itself veers toward high levels of caste and religious endogamy (or homogamy). Mixed marriages, inter-caste, inter-class marriages occur mostly where there is at least a semblance of autonomy when it comes to choosing partners (Goli et al., 2013). These marriages are often viewed as an exercising of power and feminism. In the context of religious, caste, class, and other social pressures, it is indeed a brave step. To be able to exercise any choice in the matter of a partner is almost radical in a country where child marriages and exchange of dowry are still prevalent despite being illegal (Mukherjee and Sekher, 2017).

Postfeminism in the Indian context is a complicated site. Replacing the white middle-class (and upwards) woman, we have the educated, brown, upper caste, middle class (and upwards) woman. For her, performing femininity within the bounds of culture and tradition is seen as a choice. As mentioned above, power comes from different places. However, to give up on power, while it might seem like a choice being exercised, is far from the actuality. Women who are well-placed and “choose” motherhood over a working life are encouraged to do so by social conservatism and a false valorization of childbearing. For many, to choose to stop working is a luxury that they cannot afford.

Both the series seem to fit the essence of postfeminism and most definitely seem to fit how the site of the production of postfeminism is not just in white cultures, but everywhere, even with the way heteronormativity is played out. For instance, when Rabanne (in *What the Love!*) expresses that he wants to be the damsel in a relationship, he feeds the notion of being the woman in a relationship—the damsel in distress waiting for her knight in shining armor.

While this reproduction of this stereotype of gay relationships needing someone to be masculine and someone to be feminine does no favors to the LGBT community trying hard to break out of these heteronormative standards, it also does not take into account the different pressures women are put into in a relationship or marriage where they are expected to compromise from the word “go.” They are expected to take on their husbands' last name to putting their husbands' careers before their own, not to mention being expected to lessen their engaging with their birth family.

The candidates of the shows are easy to connect with. We have met these people in our own lives—from individuals scarred by abusive relationships to children of broken marriages, and much more. It is easy to empathize with many of them. Yet, at the same time, in both shows, the male candidates, barring a couple, exhibit a lot of entitlement. When the same entitlement is shown by the women (particularly in *Indian Matchmaking*), they are seen as difficult and are expected to compromise.

Both shows have different levels of handholding for the candidates. However, how much of this is actually helping them, or for that matter, the audience viewing the show? The celebrities who are brought in on *What the Love!*, not to take away from their own lives' challenges, can hardly be called qualified to advice people who they have just met on the show. An astrologer brought in to advise Aparna on *Indian Matchmaking*, the face-reader who doesn't get a single prediction right, feeds into an archaic vision of Indian people and their relationships. However, this vision is actually closer to day-to-day reality than not. Indian jewelers thrive on having a special astrology division. There, people consult with in-house astrologers, to be told that buying gems worth several thousand rupees is the way to their salvation—both material and spiritual. At times, people who can ill-afford them go into debt at times in order to buy these jewels. Indian television has several soaps already egging on superstition and patriarchal beliefs. The question here is—do we need to have the same enforced on a reality show?

By the time the season ends, Aparna's strong stance about who she wants as a life partner is suddenly relegated to the cosmic powers of an astrologer. Nadia's need to find approval of her ethnic identity drives her toward finding an Indian (or Indian origin) partner, while trying to navigate her not being Indian enough in the eyes of other types of Indian immigrants. They are showcased as independent choices by the women. However, the complexities of identity, caste, class and privilege keep intersecting in these matches—upholding some, and pushing down others.

For many people, particularly in India, as mentioned above, wifehood could mean to not have to work menial jobs and protection from unwanted advances. It is a form of feminist agency, which someone from a higher class may never identify with. It could also be akin to caste aspirations. For others, working outside the home is emancipation. However, where middle class (and upwards) people choose to give into the more hegemonic constructs of marriage (a parade of products and wealth and giving away of the bride) even there, the notion of an unmarried woman, or someone who isn't at least seeing someone or betrothed to someone, is seen to be incomplete and, at times, a threat. Divorce rates in India are one of the lowest in the world, with just 1% of married people opting for divorce. It is often perceived to be a Western construct, and the stigma around it is such that women are often socially invalidated. Particularly for women who don't have their own means of support, it is hardly a matter of choice, despite abysmal situations on the home front. Sometimes, they feel it is just better to be separated. The stigma of divorce is way too much to handle (Deccan Herald, 2022).

In a complex cultural entanglement like India's, where parallel feminisms and postfeminisms work together, it is important to produce more socially aware, less policed reality shows instead of reifying toxic normativities, patriarchy and superstitions. These two shows are some of the first forays of Indian reality television in the English language. While they open the doors to some excruciatingly painful realities in India, they don't delve into serious conversations around it, nor do they try to break out of them much. However, whether they will learn from their mistakes and come back for further seasons with less to complain about remains to be seen.

## Author's note

DG has aimed at looking at the construction of love and marriage in modern India through the lens of two popular Netflix reality shows and how this has inadvertently pushed forward a postfeminist perspective.

## Data availability statement

The original contributions presented in the study are included in the article/supplementary material, further inquiries can be directed to the corresponding author.

## Author contributions

The author confirms being the sole contributor of this work and has approved it for publication.

## Conflict of interest

The author declares that the research was conducted in the absence of any commercial or financial relationships that could be construed as a potential conflict of interest.

## Publisher's note

All claims expressed in this article are solely those of the authors and do not necessarily represent those of their affiliated organizations, or those of the publisher, the editors and the reviewers. Any product that may be evaluated in this article, or claim that may be made by its manufacturer, is not guaranteed or endorsed by the publisher.

## Footnotes

^1^Wallace, G. (2004). *The Project Gutenberg EBook of Beethoven's Letters 1790-1826, Vol. 1 of 2 by Lady Wallace, 1866 (Alpha ed.)*. Available online at: https://www.gutenberg.org/files/13065/13065-h/13065-h.htm#let15.

^2^Throughout the paper I refer to these shows just as *Indian Matchmaking* and *What the Love!* without referring to their creators. Where necessary, I point out the episodes.

^3^This paper focuses on only cis men and women, as they are the primary subjects in the two shows in question.

^4^Indian Human Development (IHD) survey 2005.

^5^A region of modern-day Pakistan, but people who refer to themselves as Sindhi are primarily those who moved to India on the partition of India in 1947.

^6^Someone who likes being at home, and being a homemaker. Not to be confused with the meaning used in the United States of being less than average to look at.

^7^Queer relationships often strive to break down stereotypes around one partner being dominant, another submissive, one being more masculine, another more feminine. At a supermarket in Johannesburg, a till lady asked me if my husband and I were friends as she had noticed that we always seemed to be together. On telling her that we were husbands, she cooed over it, and then asked who the husband was and who the wife was. While it wasn't surprising to get this sort of question, to have it being pushed forward from within the community makes it somewhat painful.

^8^For the uninitiated, the top is considered to be more dominant or active, the bottom more submissive or passive, and a person is versatile when they can play both roles. This is a very rudimentary description of what may be sexually expected from a man who has sex with men, and is, by no means, exhaustive.

^9^Bollywood being the nickname for the Hindi cinema industry, primarily based out of Mumbai, India.
